# On the interchangeability of sea-surface and near-surface air temperature anomalies in climatologies

**DOI:** 10.1038/s41598-020-64167-1

**Published:** 2020-05-04

**Authors:** Angelo Rubino, Davide Zanchettin, Francesco De Rovere, Michael J. McPhaden

**Affiliations:** 1 University Ca’ Foscari of Venice, Department of Environmental Sciences, Informatics and Statistics, via Torino 155, 30172 Mestre, Italy; 2 NOAA/Pacific Marine Environmental Laboratory, 7600 Sand Point Way NE, Seattle, Washington 98115 US

**Keywords:** Atmospheric dynamics, Physical oceanography

## Abstract

On global and hemispheric scales, sea-surface temperature (SST) anomalies are assumed to be good surrogates for near-surface marine air temperature (MAT) anomalies. In fact, global gridded temperature datasets commonly blend SST and near-surface air temperature anomalies to overcome the lack of geographically homogeneous and reliable MAT observations. Here, we show that SST and MAT anomalies differ regarding crucial statistical properties such as multiannual trends and probabilistic distributions of daily and monthly averages. We provide evidence of the lack of interchangeability from an array of moored buoys in the tropical Pacific Ocean. We identify statistically significant discrepancies between SST and MAT anomalies for single as well as groups of such buoys. Thus, caution is required when characterizing and interpreting MAT variability through SST observations, especially at shorter than decadal timescale.

## Introduction

Near-surface air temperature observations over the oceans - the large majority of the Earth’s surface area - are relatively scarce. Until a few decades ago, they were limited to coastal areas and major shipping routes^1^. Nowadays, several monitoring programs are active in different oceanic regions. Among them, the Tropical Atmosphere Ocean (TAO) project was initiate in the 1980s to meet the growing needs of monitoring, understanding and predicting El Niño events and related phenomena^2^. The TAO array (now referred to as TAO/TRITON) consists of about 70 moorings covering the Tropical Pacific Ocean that currently provide multidecadal time series of surface and near-surface oceanographic and meteorological variables, including sea-surface temperature (SST) and near-surface marine air temperature (MAT).

Several attempts have been undertaken by the climate research community to merge information from the poorly observed early decades to the better observed recent decades and generate spatially homogeneous global gridded temperature datasets covering the full instrumental period^3–8^. In such datasets, near-surface air temperature anomalies over land and SST anomalies are commonly blended, assuming that at the hemispheric and larger scales SST variations are good surrogates of MAT variations^9,10^. In this sense, many studies have reported on the similarity between MAT and SST anomalies on large (global and hemispheric) spatial scales^9–20^. A comparative analysis of SST and satellite-measured MAT shows that, notwithstanding a distinct difference between both variables, about 80% of the variance of one is captured by the other^21^. Then, night-time MAT (nMAT) estimates are used to identify and remove SST biases to construct climate data records of SSTs in SST datasets^22^. Accordingly, during the past few decades, global gridded surface temperature datasets over the ocean have been extensively used to put oceanic climate variability in the context of global climate change^23^. Still, some studies point to issues potentially affecting the comparability between SST and MAT at large (even global) scales. Among them, MAT-SST differences observed in the tropics and in other regions of the world ocean can be non-stationary^24^; there are differences already across available observed global SST datasets^25^; the *in situ* surface marine climate observing system has been deteriorating in recent years^26^; observation-simulation comparisons on global-average surface temperature trends are affected by biases likely due to the SST-MAT blending in observations^27^. Even fewer studies have been devoted to addressing interchangeability between MAT and SST anomalies on small (local and regional) spatial scales.

In this study, we discuss the interchangeability between SST and MAT anomalies on local and regional spatial scales and on temporal scales ranging from daily to interdecadal. Interchangeability is intended here as the viability of exchanging between SST and MAT data, which stems from both variables featuring indistinguishable temporal evolution of their statistical properties. Our analysis focuses on daily as well as monthly mean estimates of SST and MAT acquired by TAO buoys. We aim to answer the following scientific questions: Are the daily anomalies of local MAT and SST different and, if so, how do these differences affect the interchangeability of monthly average anomalies? Do local differences between SST and MAT generate spatial patterns that can be attributed to known phenomena of large-scale climate variability? Do observed MAT and SST data contain significantly different interannual to interdecadal trends?

### Interchangeability of local anomalies of SST and MAT

**Figure 1:**
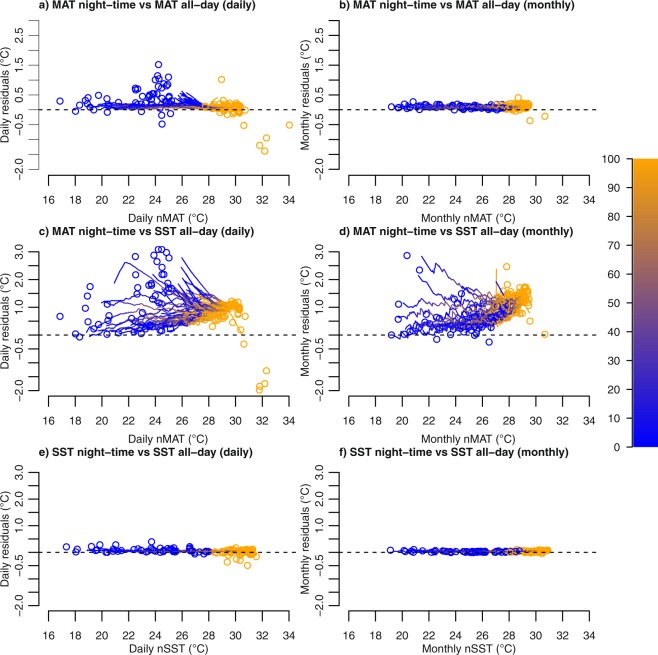
Residual Quantile-Quantile (rqq) plots of daily (**a,c**) and monthly (**b,d**) average temperature data collected by the TAO buoys at a hourly and 10-minute frequency. (**a,b**) nMAT (night time: from 20:00 to 03:50 of the next day) versus all-day MAT (all-day: from 04:00 to 03:50 of the next day); (**c,d**) nMAT versus all-day SST; (**e,f**) nSST versus all-day SST. The colour scale represents the range of quantiles. Blue and orange circles represent minima (0^th^ percentile) and maxima (100^th^ percentile). The dashed line indicates the perfect match between both variables.

The present analysis focuses on monthly average of nMAT instead of monthly average of all-day MAT in order to exclude any potential effect of known radiative heating on moored buoy air temperature sensor (see also methods). MAT and nMAT TAO data appear to be interchangeable: the residual quantile-quantile (rqq) plots of all-day MAT versus nMAT scatter around a mostly horizontal line with slight, rather constant positive residuals (Fig. 1a, see also Supplementary Figure S1). This implies that the absolute values of both variables differ by a mostly constant amount, hence that their anomalies are practically indistinguishable, hence exchangeable. This behaviour is even more pronounced at the monthly time scale, where a constant mean difference of about 0.1 °C is clearly apparent (Fig. 1b). Therefore, the radiative heating error seems not to affect the interchangeability between all-day MAT and nMAT, especially at the monthly time scale. Nonetheless, we use nMAT in the following analysis of SST-MAT interchangeability.

Unlike between MAT and nMAT, we observe substantial deviations from a horizontal line in the rqq plots when daily-average nMAT data are compared with the corresponding SST data (Fig. 1c), meaning that the interchangeability between daily SST and nMAT does not hold generally at the daily time scale. In all buoys we identify predominantly positive residuals, indicating that SSTs are virtually always warmer than nMATs. Different buoys display different, and in several cases even contrasting, behaviours as illustrated by the spread of the lines in Fig. 1c: SST-nMAT residuals can either grow or decrease with nMAT values, often exhibiting a markedly nonlinear shape. Note that a vertical displacement of the rqq curves from the horizontal zero line would still imply interchangeability between nMAT and SST anomalies; instead, any shape that is not linear or strictly horizontal implies lack of interchangeability. The rqq plots tend to converge toward a residual of about 1 °C above SST values around 29 °C. A plausible physical mechanism explaining this convergence is connected with the emergence of deep atmospheric convection only for SST exceeding a critical threshold value in the range 27–28 °C^28,29^ and its increasing efficiency with increasing SSTs. The rqq plots further reveal that, in many buoys, SSTs have larger variance than nMAT, as shown by residuals predominantly increasing with SST. However, there are also several buoys where the variance is larger in nMAT than in SST. The lack of interchangeability observed at the daily timescale holds for monthly estimates as well, with an even clearer non-linear dependence of the SST-nMAT difference on the background temperature (Fig. 1d). There are also no appreciable differences between night-time SST and all-day SST (Fig. 1e,f). Therefore, possible differences between SST and nMAT data are virtually independent from the different temporal periods considered for calculating the two variables.

**Figure 2:**
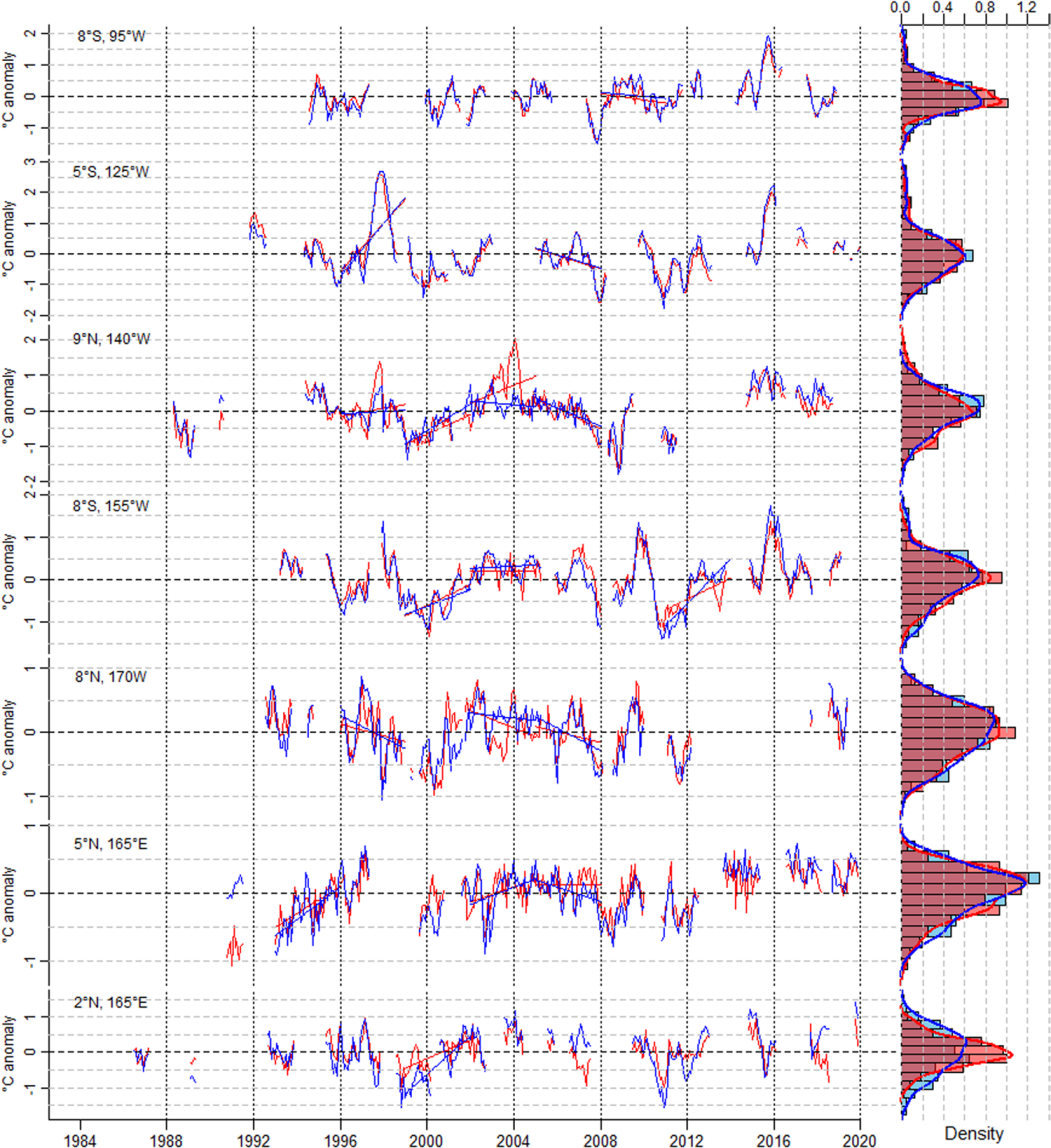
Monthly mean time series of deseasoned SST (blue) and nMAT (red) anomalies for selected TAO buoys (left panels) with associated frequency histograms and empirical probability distributions (right panels). Histograms and distributions are determined by binning the data into 14 equal-size bins. Data are calculated from daily, hourly and 10-min measurements. Straight lines indicate linear trends calculated over 3-year selected periods. Trends are not shown for those 3-year periods in which 15% or more of the data are missing.

The interchangeability between SST and nMAT varies noticeably between the different buoys and, within a single buoy, as time elapses. Figure 2 shows a few examples of this behaviour. Anomalies of the data collected at buoy 8°S, 95°W, in the eastern Pacific, exemplify an overall good superposition between deseasoned SST and nMAT, with differences never exceeding a few percent of the total variability (note that in this buoy both variables display a strong seasonal cycle, not shown). An analysis of the associated frequency distributions reveals that anomalies of both variables are distributed unimodally, but with nMAT exhibiting a higher relative amplitude of the peaks and slightly smaller variance compared to SST. Still in the eastern Pacific, in the buoy at 5°S, 125°W (where a similar predominance of the seasonal cycle on total variability occurs, not shown) nMAT and SST anomalies display occasionally larger differences, but their frequency distributions overlap well. At 9°N, 140°W, in the central Pacific, both time series often largely diverge and occasionally display even anomalies of opposite sign. We note that the relative range of variability changes through time: During some periods nMAT varies more strongly than SST, while in others SST shows fluctuations of larger amplitude than nMAT. The resulting distributions feature similar variances but markedly different kurtosis. A similar behaviour is seen also in the buoy at 8°S, 155°W. Buoys at 8°N, 170°W and at 5°N, 165°E, in the western Pacific, exemplify cases for which high-frequency fluctuations in SST and nMAT anomalies differ substantially, as reflected in the higher moments defining the resulting distributions. Still in the western Pacific, the buoy at 2°N, 165°E further exemplifies how SST and nMAT data can temporarily show anomalies of opposite sign (e.g., around 2007) and distributions differing in both variance and skewness. At a glance, often higher values of SST than nMAT are observed during warm periods. Accounting for the different geographical location of the buoys, we note a zonal pattern in the SST-MAT relation: Distributions of monthly nMAT absolute values tend to have a smaller variance than the corresponding SST distributions toward the western/central Pacific whereas both distributions tend to superpose to each other toward the eastern Pacific, likely due to dominance of a strong seasonal cycle there (not shown).

In summary, from the above analyses it seems clear that, generally, MAT and SST, and their deseasoned anomalies, cannot be considered as locally interchangeable, as far as their temporal evolution and statistical distributions are concerned. At the local scale, anomalies can even show opposite signs, while differences in anomalies can substantially exceed 1 °C. We remark that our conclusion from these examples about the lack of interchangeability between SST and MAT applies for the whole TAO dataset (not shown).

### Multiannual trends in SST and MAT

Figure 2 suggests also that multiannual trends emerging in the deseasoned SST and nMAT time series can markedly differ (see, e.g., buoy 9°N, 140° W around 2004). Long-term discrepancies in SST and nMAT trends become clear when the difference between monthly time series of both variables is plotted (Supplementary Figure S2). Such discrepancies are exemplified by the prominent variations observed in the SST-nMAT residuals throughout most of the observational period in buoy 8°S, 155°W, and in the warming trends during the period 1998-2003 in buoys 5°N, 165°E and 2°N, 165°E.

**Figure 3:**
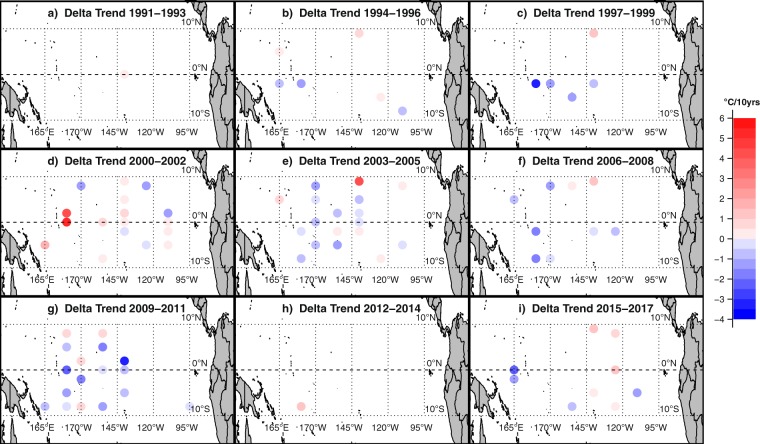
Map of the difference in the linear trend between SST and nMAT in the monthly deseasoned TAO data for selected 3-year periods between 1991 and 2017. For each 3-year period, only buoys having at least 85% of data coverage are considered. Data for a given month and buoy is considered if both nMAT and SST estimate is available. Positive values indicate that SST trends are more positive than the corresponding nMAT trends, and vice versa. The original TAO data are acquired at a daily, hourly and 10-minute frequency.

Figure 3 maps the differences in the observed multiannual trends of SST and nMAT for a selection of 3-year periods between 1991 and 2017. Such trends obviously are strongly influenced by variations in the El Nino- Southern Oscillation (ENSO), since warm and cold ENSO phases typically recur with a time scale of 4-5 years^30^. Multiannual trend differences often exceed 1 °C per decade in their absolute value. Apart from some clear outliers that may reflect local and temporary data issues such as at buoy 9°N, 140°W in 2003–2005, the patterns display a recognizable large-scale spatial structure. The persisting predominance of bluish colours in the southwestern area indicates that nMAT trends are typically more positive than SST trends. In the frame of a warming scenario, this result is in agreement with the empirical evidence stressed before, that SST warming is limited by the onset of deep atmospheric convection above a threshold value. Again, some areas experience changes of sign in the difference between SST-nMAT multiannual trends, as shown for instance in the south-central equatorial Pacific, changing from positive in the years 2003–2005 to negative in the years 2009–2011. In some periods, the spatial distribution of positive and negative trend differences seems to allow one to link them to large-scale phenomena evolving over the investigated area. For instance, the period 2000–2002, characterized by a transition from cold to warm ENSO phase, features positive trend differences in almost all the equatorial Pacific. Instead, the period 2003–2005, mostly featuring a warm ENSO phase, exhibits predominantly negative trend differences in the central-western equatorial Pacific. Overall, once again, the general interchangeability between SST and MAT seems not to hold when multiannual trends are considered.

### Comparison between TAO and gridded data

**Figure 4:**
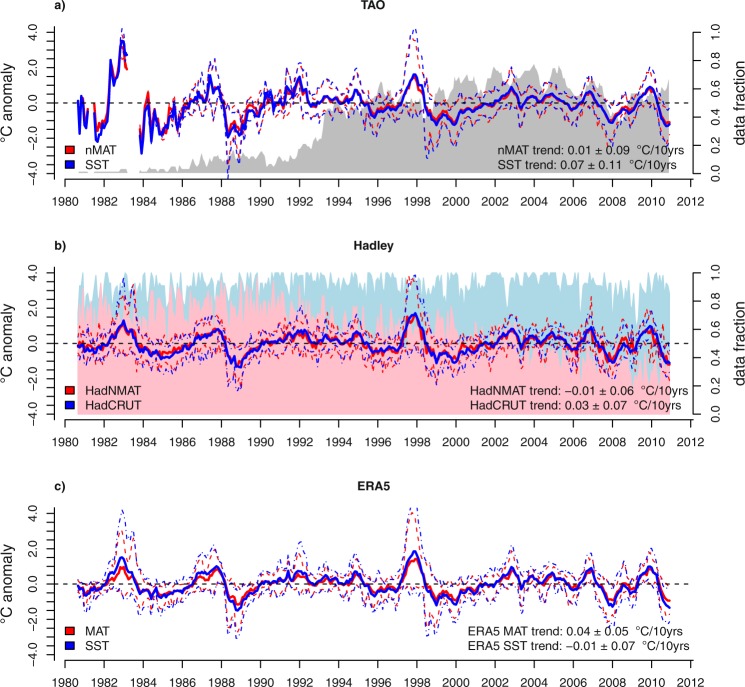
Comparison between spatial-average deseasoned SST and marine air temperature (MAT) estimates over the equatorial Pacific region for the period 1980-2010 from different datasets, with associated linear trends. Thick continuous line: mean; dashed lines: 5-95 percentile range of available individual observations at each time step. All data are interpolated to the TAO grid data (see methods). The shading in panels a and b reports the temporal evolution of data availability for TAO data and Hadley data, defined as follows: for the TAO data (grey), the data fraction refers to the number of buoys generating 10-minute, hourly and daily data over the total number of buoys comprising the TAO array. For Hadley data (pink: HadNMAT; pale blue: HadCRUT), the data fraction refers to the number of grid points with actual measures over the total number of grids in the considered spatial domain (see methods). Trends are calculated at the 95% confidence level.

So far, we have explored the SST-MAT interchangeability based on assessments from individual TAO buoys at selected mooring locations. However, spatially aggregated estimates as well as estimates from different data sources are commonly used in climate research. Therefore, it is instructive to assess the viability of interchanging aggregated monthly-mean SST and MAT estimates from TAO buoys and other sources, for instance by spatially averaging data over the equatorial Pacific region. Figure 4 illustrates the overall good superposition of deseasoned SST and MAT data in various datasets (see methods). Comparison between different products further indicates that, except for the earliest years, when few TAO data are available (see Supplementary Figure S3), the deseasoned MAT and SST evolutions appears to be very similar among the different datasets (see Supplementary Figure S4). Interdecadal trends of each of SST and MAT pair among the different datasets appear to be overall consistent within the associated uncertainties (Fig. 4). More importantly for our assessment of long-term behaviour of spatially-integrated values, we note that despite differences in the estimates of the interdecadal trend between SST and MAT exist within each of the considered datasets, these generally overlap within the respective standard error estimates (Fig. 4). Supplementary Table S1 provides additional evidence for the variety of trends detectable at interannual and decadal time scales in the considered products and variables. Note, in some periods, the emergence of large uncertainties. Overall, there are noticeable differences in the best estimates of the trend component included in regional SST and MAT time series derived from local instrumental (TAO), gridded observational (Hadley) and reanalyses data (ERA5). However, such differences tend to vanish the longer the considered time period, i.e., agreement increases when the analysis passes from multiannual to decadal and interdecadal trends. This result again suggests caution when analysing gridded products that interchange MAT anomalies with SST anomalies, especially at shorter than decadal time scales.

### Concluding remarks

This work aimed at assessing the validity of the assumption that MAT and SST anomalies are interchangeable at the local and regional spatial scales, and over temporal scales ranging from daily to interdecadal. Our results for the equatorial Pacific region indicate that MAT and SST cannot be considered as interchangeable as far as their temporal evolution and statistical distributions are concerned, with potentially significant repercussions on the estimation of multiannual variability. This lack of interchangeability between SST and MAT seems to hold on regional scales for datasets of different characteristics, including raw instrumental measurements, derived gridded observations and data reanalyses. We therefore suggest caution when analysing global surface temperature products locally and regionally on short time scales.

## Data and methods

### Data

We make use of MAT and SST data acquired by moored buoys constituting the Tropical Atmosphere Ocean (TAO) array maintained by the National Oceanic and Atmospheric Administration (NOAA) of the United States of America. TAO originated in the mid-1980s, was completed in 1994^31^, and became known as TAO/TRITON, a partnership between the Japan Agency for Marine Earth Science and Technology (JAMSTEC) and NOAA in 2000. The TAO/TRITON array is comprised of approximately 70 moorings deployed in the Tropical Pacific Ocean between 8°N and 8°S, 95°W and 137°E. An update of the mooring technology in 1996 (deployment of the Next Generation system) made possible the collection of 10-minute data, which is used in the study at all available latitudes of the following longitudinal locations in the TAO portion of the array (from east to west): 95°W, 110°W, 125°W, 140°W, 155°W, 170°W, 180°W, 165°E. Supplementary Figure S3 illustrates the length of buoy records considered in this study, which includes daily, hourly and 10-minute data. Data are publicly available and can be retrieved from https://www.pmel.noaa.gov/tao/drupal/disdel/. The data are subject to extensive quality check. For an overview please see: https://www.pmel.noaa.gov/gtmba/sampling.

Air temperature is sampled at 2 m height by a resistance temperature recorder at a 0.01 °C resolution, with an accuracy of ±0.02 °C. SST measurements are retrieved 1 m below the surface of the ocean by a thermistor with a resolution of 0.001 °C and an accuracy of ±0.02 °C^32^ (https://www.pmel.noaa.gov/gtmba/sensor-specifications). Details containing estimates of uncertainty concerning TAO measurements can be found, e.g., in Castro *et al*., 2012^33^ and in Anderson and Baumgartner, 1998^34^. High-frequency 10-minute measurements are recorded internally; every 6 months to 1 year, these data are retrieved, processed, archived, and made available publicly. Hourly (since the deployment of the Next Generation system) and daily data are telemetered in near-real-time to PMEL offices.

We pre-processed the TAO data as follows. Firstly, we subsample the dataset in order to exclude poor quality data. To this purpose, we refer to the quality codes reported for the dataset (https://www.pmel.noaa.gov/gtmba/data-quality-control) and select only quality code 1 (highest quality) and 2 (default quality) data. Supplementary Figures S5 and S6 illustrate the distribution of quality in the considered dataset. Then, only MAT night-time measurements (between 8 pm and 3.50 am local time the next day) are retained because of radiative heating error in the daytime measurements due to the use of the naturally ventilated technology during daytime^34^. All-day daily MAT values are defined as the average of the measurements over a full 24 hr time period (from 04:00 to 03:50 of the next day) and are utilised for comparison with the correspondent daily nMAT values. SST data are not subjected to any pre-processing. Panel a) of Fig. 1 suggests a good affinity between TAO nMAT and all-day MAT daily mean values calculated from hourly and 10-minute measures. Although TAO daily MAT data are affected by the radiative heating error, their use is of major importance for comparing SST and MAT anomalies and trends on larger temporal periods. Therefore, the period of analysis is extended back to the first 1980s to include daily TAO data.

For the calculation of monthly mean time series, the raw nMAT and SST data are averaged for each available month. Months with more than 5 non-continuous days of missing data are excluded from the following analysis. Therefore, the total number of missing 10-minute data allowed in one month is 144*5 for SST and 48*5 for nMAT while, for hourly data, the total number of missing measures allowed is 24*5 for SST and 8*5 for nMAT. Anomalies are computed for nMAT and SST monthly series considering only those months when values for both variables are available. Our analysis is based on monthly anomalies to permit a suitable comparison with the global gridded surface temperature datasets, as these datasets utilise monthly temperature anomalies. Anomalies of buoy and gridded datasets are calculated by removing, for each month of the year, the associated long-term average over the available period. Therefore, the average seasonal cycle is removed from the data.

We use the publicly available gridded temperature datasets of nMAT (HadNMAT^8^, version 2.0.1.0) and SST (HadCRUT^9^, version 4.6.0.0). Both datasets comprise observations gridded on a 5° × 5° grid with global coverage. The HadNMAT dataset covers the period spanning from the late 19^th^ century to 2010 while HadCRUT is currently updated on a monthly basis. Furthermore, we utilise the Absolute^35^ temperature dataset providing the mean temperature climatology, thus allowing the computation of HadCRUT absolute values for the 1980–2010 period and the associated mean annual cycle.

Reanalysis output is from the ERA5 reanalysis provided by ECMWF^36^.

For all gridded datasets, we only use data relative to the TAO geographical area, specifically defined as spanning from 10°S to 10°N latitude and from 160°E to 90°W longitude. All gridded datasets are spatially bilinearly interpolated to the TAO grid to improve comparability.

### Methods

In the main analysis, MAT and SST series from current datasets and for a variety of grid-points are compared to investigate whether the two variables have the same evolution and statistical properties.

We use residual quantile-quantile (rqq) plots to illustrate differences between pairs of variables^37,38^. In rqq plots, the differences between the distribution quantiles of the variable under study and those of a reference variable are plotted on the y-axis against the quantiles of the reference variable plotted on the x-axis. In this way, rqq plots emphasise the deviations between the distributions of the variable under study and of the reference variable, particularly allowing assessment of whether the climatological distribution of an estimate of interest is similar to the distribution of the target. We are thus able to identify whether the empirical quantiles for each individual ensemble member agree with the verification data sample. Plotting the residuals eases the interpretation since ideal agreement between estimated and verification quantiles leads to vanishing residuals, i.e. a horizontal line crossing the y-axis at zero. Disagreements can be easily identified. Among the possible deviations identified by rqq plots are differences in the tails of the distributions, their skewness or their means. Biases in the mean yield horizontal displacements from the expectation of vanishing residual quantiles: a constant offset in the rqq plots means that absolute values of both variables are not interchangeable, but the anomalies are. Differences in estimated and target climatological variances are seen as a positive slope in the residuals if the estimated climatological distribution is wider than the target climatology distribution, and as a negative slope if it is narrower. nMAT and SST values are further compared based on the difference between the monthly averages of both variables, hereafter referred to as “delta”.

The empirical distributions of SST and nMAT are compared for each considered buoy individually. Data distributions are inspected through density histograms and density lines. Density histograms are computed utilising a fixed number of breakpoints (15). Density lines are estimated through a Kernel algorithm.

## Supplementary information

Supplementary Material.
