# Supplementary material for: On the interchangeability of sea-surface and near-surface air temperature anomalies in climatologies
Source: Sci Rep. 2020 May 4;10:7433. doi: 10.1038/s41598-020-64167-1 (PMC7198542; doi:10.1038/s41598-020-64167-1)
Supplement: Supplementary file 1 — Supplementary Material. [file 41598_2020_64167_MOESM1_ESM.docx]

**On the interchangeability of sea-surface and near-surface air temperature anomalies in climatologies**

Angelo Rubino*^,1^, Davide Zanchettin^1^, Francesco De Rovere^1^, and Michael J. McPhaden^2^

1. University Ca’ Foscari of Venice, department of environmental sciences, informatics and statistics, via Torino 155, 30172 Mestre, Italy
2. NOAA/Pacific Marine Environmental Laboratory, 7600 Sand Point Way NE Seattle, Washington 98115

* corresponding author: [rubino@unive.it](mailto:rubino@unive.it)

**SUPPLEMENTARY MATERIAL**


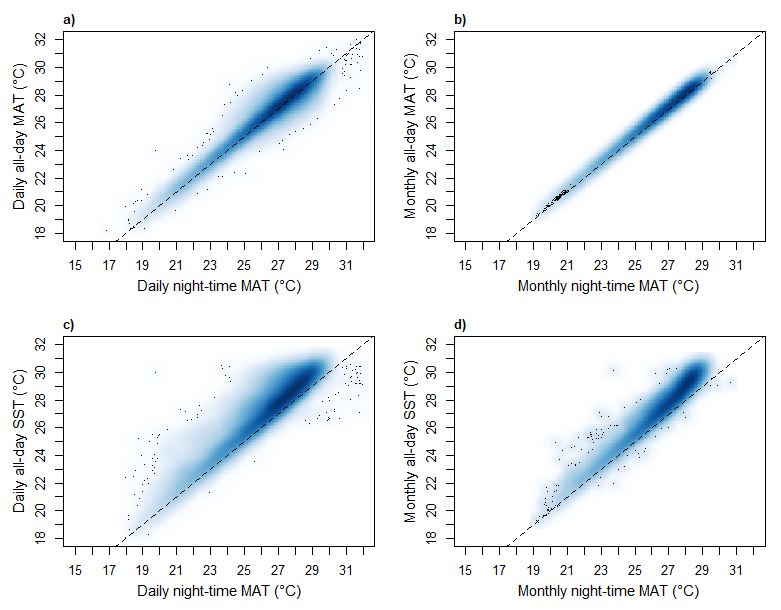


**Supplementary Figure S1** - Density plot of daily (a,c) and monthly (b,d) average temperature data collected by the TAO buoys at a daily, hourly and 10-minute frequency. a,b: night-time MAT (from 20:00 to 03:50 of the next day) versus all-day MAT (from 04:00 to 03:50 of the next day); c,d: night-time MAT versus all-day SST. Black points correspond to outliers.


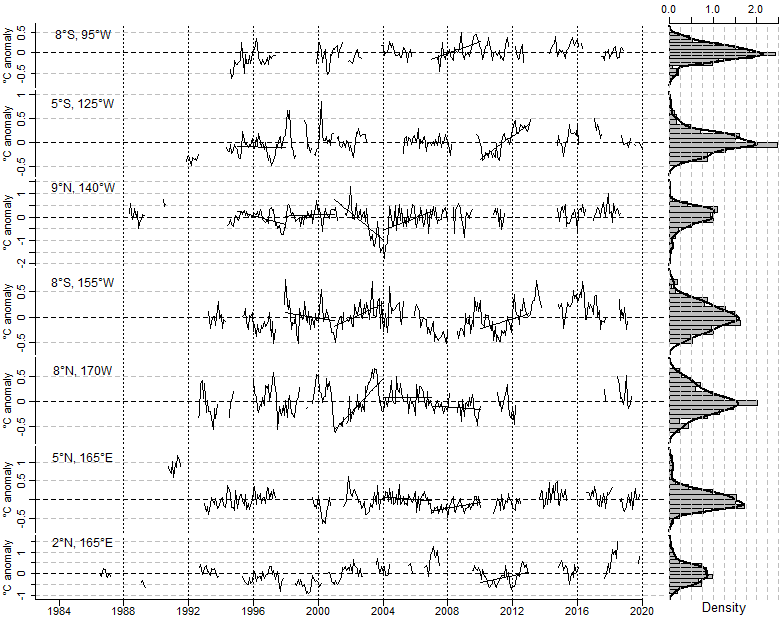


**Supplementary Figure S2** - Time series of the monthly residuals of deseasoned SST-nMAT for selected TAO buoys with associated frequency histograms and empirical probability distributions (right panels). Black horizontal lines highlight zero values, i.e., the congruence between the two variables.


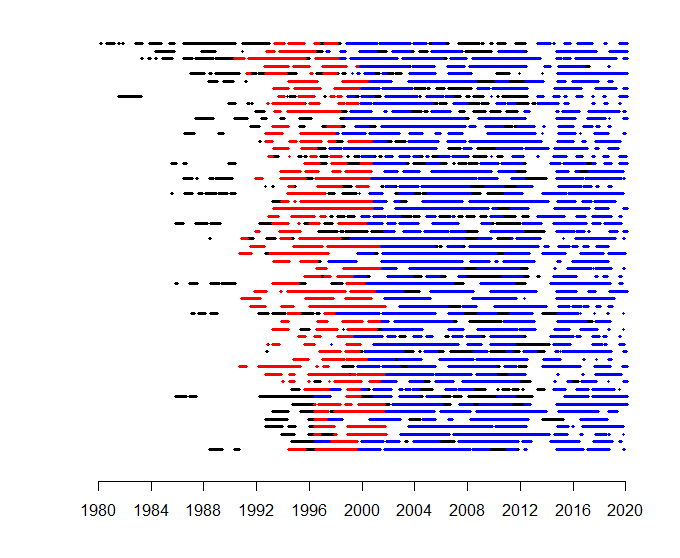


**Supplementary Figure S3** –Availability of TAO data. Each horizontal line refers to one of each of the considered TAO buoys, and identifies periods in which the buoy registers both SST and MAT. The sampling rate is colour coded: black: daily; red: hourly; blue: 10-minute.


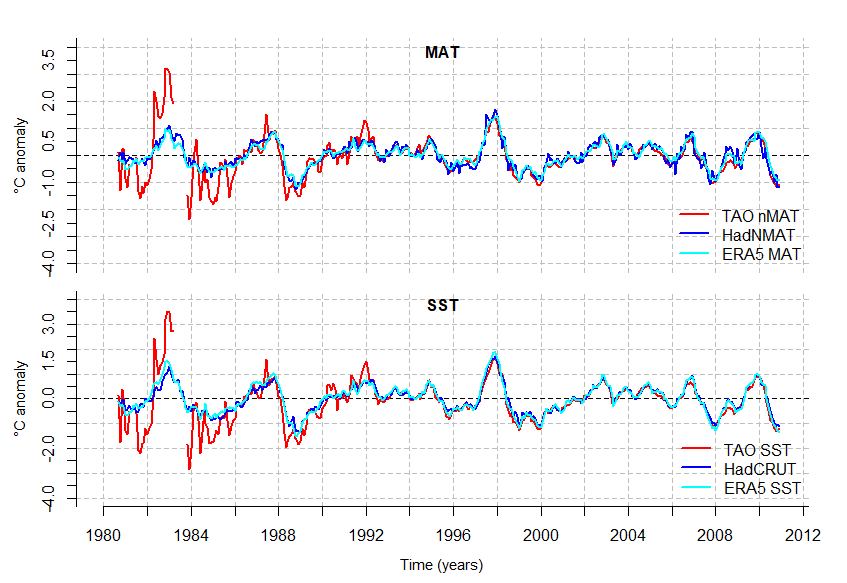


**Supplementary Figure S4** – Comparison between spatial-average deseasoned SST and MAT estimates over the equatorial Pacific region for the period 1980-2010 from different datasets. Data are the same shown in Figure 4 of the main manuscript, but SST and MAT data from different products are shown in the same panel to highlight consistency across estimates.


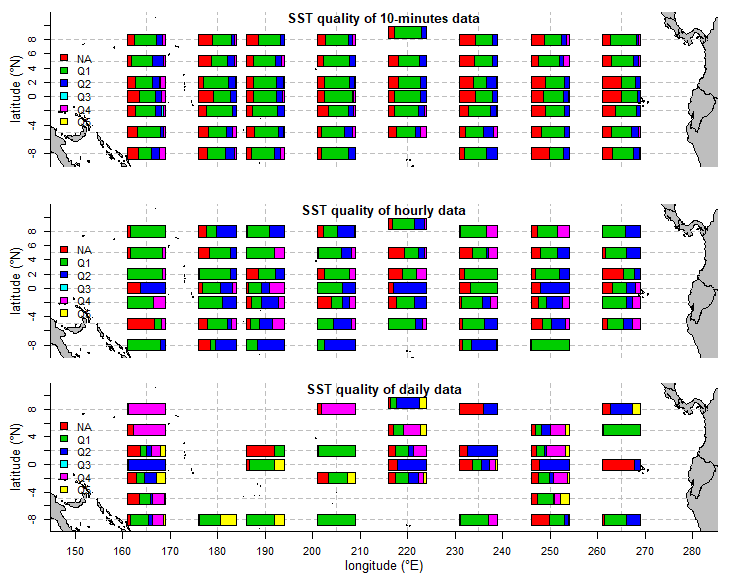
**Supplementary Figure S5** – Distributions of quality of employed SST data. Horizontal bars report with different colours the fractions of data of each quality measured by a single buoy. Quality refers to the quality codes reported at <https://www.pmel.noaa.gov/gtmba/data-quality-control>. Only data of quality code Q1 (highest quality) and Q2 (default quality) are considered in the analysis.


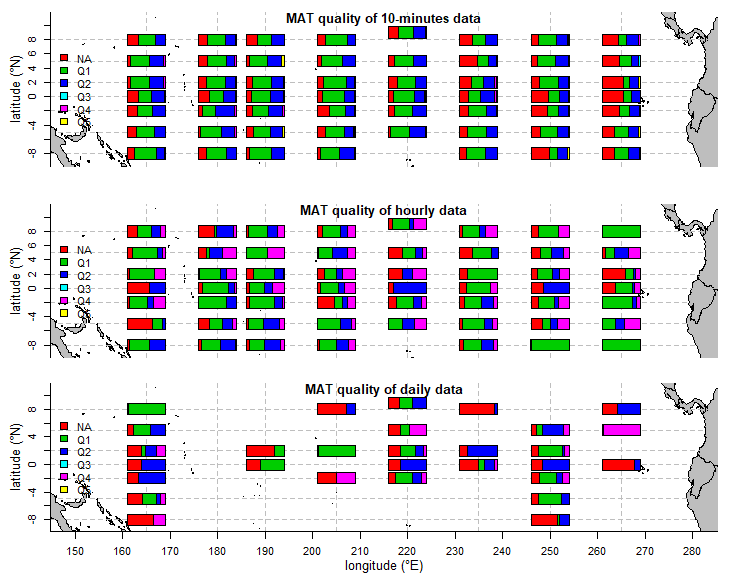


**Supplementary Figure S6** – Distributions of quality of employed MAT data. Horizontal bars report the fraction of data of each quality over the total data. Quality refers to the quality codes reported at <https://www.pmel.noaa.gov/gtmba/data-quality-control>. Only data of quality code Q1 (highest quality) and Q2 (default quality) are considered in the analysis.

|  | **TAO nMAT** | **TAO SST** | **HadNMAT** | **HadCRUT** | **ERA5 MAT** | **ERA5 SST** |
| --- | --- | --- | --- | --- | --- | --- |
| 1980-1989 | -0.66±0.85 | -0.6±0.95 | -0.47±0.14 | -0.5±0.2 | -0.3±0.33 | -0.54±0.46 |
| 1990-1999 | -0.63±0.33 | -0.85±0.38 | -0.49±0.32 | -0.51±0.34 | -0.38±0.28 | -0.6±0.37 |
| 2000-2009 | 0.07±0.26 | 0.02±0.29 | 0.13±0.27 | 0.15±0.3 | 0.16±0.25 | 0.09±0.32 |
| 1992-1994 | -0.16±1.28 | -0.98±1.41 | -0.36±0.88 | -0.69±0.92 | -0.17±0.73 | -0.39±0.93 |
| 1995-1997 | 3.8 ±1.92 | 4.45±2.16 | 4.05±2.39 | 4.36±2.31 | 3.67±1.85 | 5.1±2.41 |
| 1998-2000 | -2.87±1.89 | -3.41±2.41 | -2.83±2.03 | -4.31±2.06 | -3.50±1.88 | -3.94±2.33 |
| 2001-2003 | 1.67±1.02 | 2±1.14 | 1.65±1.16 | 2.17±1.11 | 1.88±0.91 | 2.24±1.19 |
| 2004-2006 | -0.35±1.06 | -0.29±1.15 | 0.28±1.39 | 0.11±1.30 | 0.4±1 | 0.28±1.31 |
| 2007-2009 | 3.06±1.69 | 2.97±1.94 | 3.57±1.59 | 2.91±2.01 | 2.65±1.76 | 3.28±2.16 |

**Supplementary Table S1** – Near-decadal (top rows) and interannual (3-year, bottom rows) trends calculated for a number of spatially-aggregated variables over the equatorial Pacific. Trends are calculated from the same deseasoned time series shown in figure 4, but for different and shorter time periods. All trends are expressed as °C/10yrs and are calculated at the 95% confidence level.
